# Rapid metabolic profiling of *Nicotiana tabacum *defence responses against *Phytophthora nicotianae *using direct infrared laser desorption ionization mass spectrometry and principal component analysis

**DOI:** 10.1186/1746-4811-6-14

**Published:** 2010-06-09

**Authors:** Alfredo J Ibáñez, Judith Scharte, Philipp Bones, Alexander Pirkl, Stefan Meldau, Ian T Baldwin, Franz Hillenkamp, Engelbert Weis, Klaus Dreisewerd

**Affiliations:** 1 Institute of Medical Physics and Biophysics, Westfälische Wilhelms-Universität Münster, Robert-Koch-Str. 31, D-48149 Münster, Germany; 2 Institute of Botany, Westfälische Wilhelms-Universität Münster, Schlossgarten 3, D-48149 Münster, Germany; 3 Department of Molecular Ecology, Max Planck Institute for Chemical Ecology, Beutenberg Campus, Hans-Knöll-Str. 8, D-07745 Jena, Germany

## Abstract

**Background:**

Successful defence of tobacco plants against attack from the oomycete *Phytophthora nicotianae *includes a type of local programmed cell death called the hypersensitive response. Complex and not completely understood signaling processes are required to mediate the development of this defence in the infected tissue. Here, we demonstrate that different families of metabolites can be monitored in small pieces of infected, mechanically-stressed, and healthy tobacco leaves using direct infrared laser desorption ionization orthogonal time-of-flight mass spectrometry. The defence response was monitored for 1 - 9 hours post infection.

**Results:**

Infrared laser desorption ionization orthogonal time-of-flight mass spectrometry allows rapid and simultaneous detection in both negative and positive ion mode of a wide range of naturally occurring primary and secondary metabolites. An unsupervised principal component analysis was employed to identify correlations between changes in metabolite expression (obtained at different times and sample treatment conditions) and the overall defence response.

A one-dimensional projection of the principal components 1 and 2 obtained from positive ion mode spectra was used to generate a Biological Response Index (BRI). The BRI obtained for each sample treatment was compared with the number of dead cells found in the respective tissue. The high correlation between these two values suggested that the BRI provides a rapid assessment of the plant response against the pathogen infection. Evaluation of the loading plots of the principal components (1 and 2) reveals a correlation among three metabolic cascades and the defence response generated in infected leaves. Analysis of selected phytohormones by liquid chromatography electrospray ionization mass spectrometry verified our findings.

**Conclusion:**

The described methodology allows for rapid assessment of infection-specific changes in the plant metabolism, in particular of phenolics, alkaloids, oxylipins, and carbohydrates. Moreover, potential novel biomarkers can be detected and used to predict the quality of plant infections.

## Background

Metabolism consists of a complex network of biosynthetic pathways and comprises a series of biochemical reactions that are catalyzed by enzymes [1,2]. Plants and animals produce a remarkably diverse array of over 100,000 secondary metabolites. In contrast to the primary metabolites, secondary metabolites are normally not directly involved in growth, development, and reproduction, but partly play essential roles in the adaptation of the organisms to their environments [1-5]. The rich diversity of secondary metabolites results from a co-evolutionary process with biotic attackers. Secondary metabolites stored in various plant organs and cell compartments play important roles as *constitutive defence *against herbivores and microbial pathogens. Natural selection favors those plants that have developed distinct cellular defence responses, specifically induced and tailored against the invasion of pathogens or attack by insects [1-5]. *Induced defences *against various attackers are differentially orchestrated by a network of interacting signaling pathways.

Depending on the signaling pathway employed, the defence response may in some cases only encompass the infected tissue (*local response*) or in addition involve non-infected parts of the plant (*systemic response*), thus protecting the plant against subsequent attacks [6,7]. The phytohormone salicylic acid (SA) is mainly induced by biotrophic and hemi-biotrophic parasites and initiates and controls both the systemic response and the hypersensitive response (HR) [5,8-10]. The HR is a type of programmed cell death in which the plant sacrifices a few cells immediately surrounding the attacked cell, thereby restricting pathogen growth [8-10]. For the proper development of a HR, the SA-signaling pathway includes feedback loops which integrate other defence-associated signals, such as (i) jasmonic acid (JA) [11,12], (ii) ethylene (ET) [13], and (iii) nitric oxide (NO) [14-16]. Other metabolic events, which are important in the execution of HR include reinforcement of the cell wall [17], phytoalexin production [18], expression of pathogenesis-related proteins [19], reactive oxygen species (ROS) generation [17,20], and the readjustment of the primary metabolism, that includes sugar accumulation and signaling [17,20]. The overall signature of the complex metabolic and signaling networks in the plant determines the pattern of defence-related metabolome changes and, hence, eventually the outcome of the plant - pathogen interaction [1,8,9,12].

Different methods have been developed for the analysis of multiple metabolites during the plant defence response, such as fluorescence microscopy [21-24] and mass spectrometry (MS) [25-30] Because of the high complexity of the plant metabolome, hyphenated MS-based methods are mostly preferred, *e.g*. the coupling of a mass spectrometer with gas chromatography (GC) [25-27] or liquid chromatography (LC) [28-30]. By adding reference compounds, these techniques allow the absolute quantification of individual metabolites [27-30]; however, the employment of these hyphenated methods requires cumbersome sample preparation processes [25-30].

Matrix-assisted laser desorption ionization mass spectrometry (MALDI MS) has been increasingly employed in metabolomic studies. Advantages of this analytical method are that first limited effort is required for sample preparation and second the method lends itself to high throughput analysis [31-35]. A few reports have described the utilization of ultraviolet (UV) and infrared (IR) lasers for the analysis of plant metabolites directly from tissue [32,36-40]. This is possible because laser energy in the UV or IR region can be absorbed directly by different biomolecules in the tissue. If the concentration of absorbing biomolecules is high enough, they can function as an endogenous matrix for the MALDI process. IR lasers with an emission wavelength around 3 μm, such as an Er:YAG laser emitting at 2.94 μm or an optical parametric oscillator (OPO) laser, make use of O-H containing biomolecules (*i.e*. mostly water molecules) as an endogenous matrix via excitation of the O-H bond stretch vibrations [41]. In the following, we will use the abbreviation "IR-LDI" instead of "IR-MALDI" to account for the fact that only endogenous biomolecules are used as energy absorbers.

While direct tissue analysis by mass spectrometry generally does not allow for absolute quantification of molecular concentrations, a semi-quantitative analysis may be possible by recording the changes in signal intensities of individual compounds, for instance in response to a treatment. A particularly elegant method to retrieve information about even small changes of the molecular profiles in a complex system (a LDI mass spectrum may contain hundreds of ion signals) is the use of principal component analysis (PCA) [42,43]. PCA is a multivariate data analysis which can be used to reduce the complexity of large amounts of detectable ion signals to a small number of independent (orthogonal) component vectors. Hence, in the PCA the individual identity as well as the absolute intensity of an ion signal are not as relevant as the mass spectral profile as a whole [42,43].

With respect to the mass analyzer, for the analysis of rough and electrically non-conducting surfaces (such as leaf tissue), orthogonal time-of-flight (oTOF) mass spectrometers have been found to provide particularly beneficial properties [40,44,45]. This is because these instruments decouple the ion formation process from the time-of-flight measurement and thereby provide a high and constant mass accuracy, independent of the exact morphology of the investigated sample [40,44,45]. The combination of IR-LDI and oTOF MS also provides particularly soft desorption/ionization conditions that produce a minimum of analyte ion dissociation [44,46]. The analysis of a wide range of metabolites directly from plant tissues - *e.g*. lipids (phospholipids, sulfates, triglycerides), carbohydrates (oligosaccharides), glycosidic flavonoids, and alkaloids - has been reported using this type of instrument [40].

Here, we used direct IR-LDI-oTOF mass spectrometry to simultaneously profile a large number of metabolites of the tobacco plant *Nicotiana tabacum *(Samsun, *SNN*), generated during development (1 to 9 hours post infection) of the HR against the oomycete *Phytophthora nicotianae*. A multivariate analysis - principal component analysis (PCA) - was used to reduce the large and complex LDI MS data to one- or two- dimensional data sets. The statistical analysis was compared with an established indicator of the biological response of the plant - pathogen system, dead cell count analysis, and an established method for studying plant defence responses, *i.e*. quantification of defence-related phytohormones using liquid chromatography in combination with electrospray ionization tandem mass spectrometry (LC-ESI MS/MS) [28-30]. We conclude that IR-LDI MS metabolome profiling in conjunction with PCA can be a powerful tool to obtain information about plant defence cascades and to monitor the intensity and progression of the infection processes.

## Results and Discussion

Although extensive data exists describing the role of various secondary metabolites (such as SA) during the hypersensitive defence response in pathogen-plant interaction [5,8-12], much remains to be learned about the response as a whole.

In a classical approach, it would be possible to identify the different signal cascades that are activated in the leaf during the infection by quantifying "key" metabolites for each sample treatment conditions (*i.e*. blank, placebo, and infection). However to reliably quantify these compounds, an internal standard would be required for each metabolite of interest to compensate for the matrix effects that originate from the inherent amount of variability associated with biological samples. Thus, the quantification approach is suitable for the verification of a hypothesis but is not recommended as the first step for any plant study when not much is known about the occurring cellular processes, as it is the case here.

**Figure 1:**
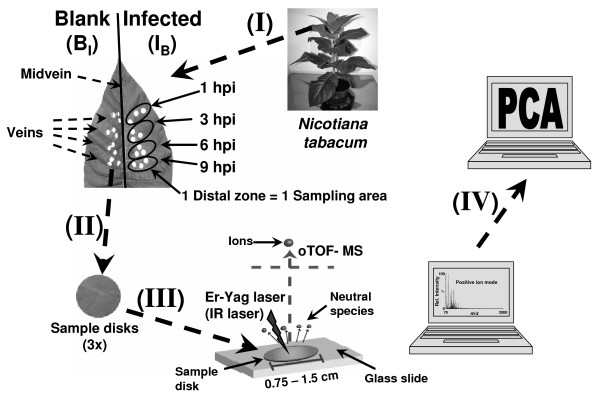
**Workflow of the combined IR-LDI mass spectrometry - principal component analysis of plant metabolites**. Schematic workflow of the analysis: (I) sample treatment, (II) sample collection, (III) direct IR-LDI-oTOF MS measurement, (IV) principal component analysis. The lower cuticle of the leaf is removed using adhesive tape and disks are placed upside down on a glass slide. The glass slide is mounted in a custom-made sample plate holder and transferred into the laser mass spectrometer for measurement. The nomenclature employed to identify the sample type is explained in the text.

Relative up- or down-regulation of metabolic cascades may be followed by employing a semi-quantitative approach. Here, we applied this method and used the earliest measurement time point obtained for each sample treatment (*i.e*. 1 hpi) as reference point. This way, we can compensate for non-biological effects (*e.g*. systemic analytical errors caused by changes in the growth environment) which could influence the metabolic response. The general procedure and experimental workflow of the analysis is depicted in Figure 1; see Materials and Methods for details.

The accessible mass range of the matrix-less LDI MS approach is limited to about 1000 *m/z *units. Because no matrix was employed in these measurements, the mass spectra are fully devoid of the matrix-derived chemical background, which can severely complicate standard MALDI MS analysis of small molecules. Moreover, signals corresponding to other relative abundant biological substances - such as peptides - also are not produced. The IR-LDI-oTOF MS measurements were performed in both positive and negative ion modes from differently treated leaf samples: zoospores infiltrated (infected, I), tap-water infiltrated (placebo, P) and untreated (blank, B), and at time points of 1, 3, 6, and 9 hpi (see Material and Methods for details on the sample preparation procedure). As an example, Additional file 1 shows the positive and negative ion mode mass spectra for the region between *m/z *100 to 500 obtained using IR-LDI-oTOF MS for two samples, infected (I_P_) and placebo (P_I_), harvested at 6 hpi from a *SNN *tobacco leaf. A full list of all consistently detected ions is provided in Table 1.

**Figure 2:**
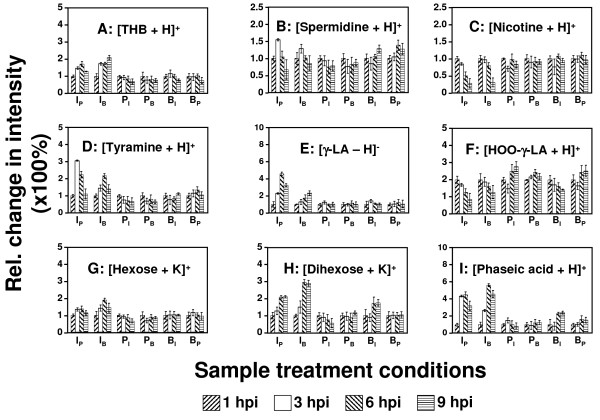
**IR-LDI-oTOF MS analysis of metabolites (time-profiles)**. Metabolic time-profiles of selected metabolites in *Nicotiana tabacum *(cv. *SNN*) samples that were subjected to different treatments: (I) infected, (P) placebo, and (B) blank (for detailed explanation of the nomenclature employed, see text). IR-LDI MS signal intensities of the molecular ions (see Table 1 for ion type) were normalized to the value obtained at 1 hpi. THB (tetrahydrobiopterin); γ-LA (γ-linolenic acid); HOO-γ-LA (peroxidated-γ-linolenic acid).

Figure 2 displays the time-profiles for a subset of 9 example metabolites, as derived from the IR-LDI MS analysis of differently treated samples; data are normalized to the signal intensity obtained at 1 hpi. The chemical assignments (identities) for these 9 metabolites and several other compounds (Table 1) could be made with reasonable confidence on the basis of precise mass measurement, the isotope ratios of the corresponding signals, and metabolic databases (see Material and Methods for details on the identification of metabolites).

**Table 1:** List of ion signals detected by direct IR-LDI-oTOF mass spectrometry in leaf samples of *Nicotiana tabacum *in (A) positive and (B) negative ion mode.*

A: Positive ion mode					
#	Obs. Mass (*m/z*)	Chemical Formula	Theor. Mass (*m/z*)	Δm (ppm)	Tentative Assignment*
a1	104.107	[C5H14NO]^+^	104.107	0.0	choline
a2	122.095	[C8H11N + H]^+^	122.097	16.4	phenethylamine
a3	132.112	[C5H13N3O + H]^+^	132.113	7.6	N-carbamoylputrescine
a4	138.091	[C8H11NO + H]^+^	138.092	7.2	tyramine
a5	146.167	[C7H19N3 + H]^+^	146.165	13.7	spermidine
a6	163.122	[C10H14N2 + H]^+^	163.123	6.1	nicotine or anabasine
a7	192.172	[C13H21N + H]^+^	192.175	15.6	
a8	219.027	[C6H12O6 + K]^+^	219.027	0.0	hexose
a9	242.126	[C9H15N5O3 + H]^+^	242.125	4.1	tetrahydrobiopterin
a10	251.221	[C14H26N4 + H]^+^	251.223	8.0	
a11	266.185	[C17H25N + H]^+^	266.186	3.8	
a12	269.170	[C17H20N2O + H]^+^	269.165	15.8	
a13	281.140	[C15H20O5 + H]^+^	281.138	6.0	phaseic acid
a14	311.221	[C18H30O4 + H]^+^	311.221	0.0	hydroperoxyoctadecatrienoic acid
a15	318.153	[C14H23NO7 + H]^+^	318.155	6.3	glycosylated-retronecin or [tyramine + hexose]^1^
a16	325.236	[C19H32O4 + H]^+^	325.237	3.1	methyl-hydroperoxyoctadecatrienoate
a17	343.186	[C19H28O4 + Na]^+^	343.189	8.7	
a18	355.153	[C19H24O5 + Na]^+^	355.152	2.8	gibberellin 20
a19	361.211	[C19H33ClO4 + H]^+^	361.215	10.0	methyl-chloro-hydroperoxyoctadecatrienoate
a20	381.079	[C12H22O11 + K]^+^	381.080	2.6	dihexose
a21	399.085	[C12H24O12 + K]^+^	399.091	15.0	[hexose + hexose]1
a22	431.222	[C23H30N2O6 + H]^+^	431.218	9.3	
**B: Negative ion mode**					
**#**	**Obs. Mass (*m/z*)**	**Chemical Formula**	**Theor. Mass (*m/z*)**	**Δm (ppm)**	Tentative Assignment*
b1	103.004	[C3H4O4 - H]^-^	103.004	0.1	malonic acid
b2	105.019	[C3H6O4 - H]^-^	105.019	2.2	glyceric acid
b3	127.016	[C4H4N2O3 - H]^-^	127.014	15.7	
b4	133.014	[C4H6O5 - H]^-^	133.014	1.3	malic acid
b5	135.029	[C4H8O5 - H]^-^	135.029	1.9	threonate
b6	146.046	[C5H9NO4 - H]^-^	146.045	3.0	
b7	173.008	[C6H6O6 - H]^-^	173.009	3.6	dehydroascorbic acid
b8	174.019	[C6H7O6 - H]^-^	174.017	11.5	mono-dehydroascorbic acid
b9	179.055	[C6H12O6 - H]^-^	179.055	1.6	hexose
b10	191.053	[C7H12O6 - H]^-^	191.056	16.6	quinic acid
b11	197.062	[C13H10O2 - H]^-^	197.060	10.3	
b12	215.031	[C6H12O6 + Cl]^-^	215.032	4.8	hexose
b13	225.057	[C10H10O6 - H]^-^	225.061	17.8	chorismic acid
b14	226.995	[C6H8O7 + Cl]^-^	226.996	1.8	citric acid
b15	239.073	[C15H12O3 - H]^-^	239.071	8.4	
b16	255.233	[C16H32O2 - H]^-^	255.232	3.0	palmitic acid
b17	267.071	[C9H16O9 - H]^-^	267.071	0.3	
b18	269.082	[C16H1404 - H]^-^	269.081	3.7	
b19	277.215	[C18H30O2 - H]^-^	277.217	6.7	γ-linolenic acid
b20	279.034	[C13H12O5S - H]^-^	279.033	1.8	
b21	279.231	[C18H32O2 - H]^-^	279.232	2.1	α-linolenic acid
b22	285.080	[C16H14O5 - H]^-^	285.076	14.8	
b23	297.045	[C16H10O6 - H]^-^	297.047	4.4	
b24	309.044	[C17H10O6 - H]^-^	309.040	12.2	
b25	310.147	[C19H21NO3 - H]^-^	310.144	8.4	
b26	313.075	[C17H14O6 - H]^-^	313.075	1.3	
b27	315.090	[C17H16O6 - H]^-^	315.090	0.9	
b28	325.044	[C9H15N2O9P - H]^-^	325.043	2.6	
b29	327.054	[C17H12O7 - H]^-^	327.051	8.7	
b30	353.088	[C16H18O9 - H]^-^	353.087	0.7	chlorogenic acid
b31	359.118	[C12H24O12 - H]^-^	359.119	2.8	[hexose + hexose]1
b32	371.111	[C20H20O7 - H]^-^	371.113	5.8	
b33	377.083	[C12H22O11 + Cl]^-^	377.084	3.9	dihexose
b34	395.095	[C12H24O12 + Cl]^-^	395.095	0.8	[hexose + hexose]1
b35	447.132	[C22H24O8 - H]^-^	447.129	8.4	
b36	457.278	[C29H42O2 + Cl]^-^	457.286	17.3	
b37	477.145	[C16H30O16 - H]^-^	477.145	0.5	

*Assignment of chemical composition is based on precise mass measurements, comparison of isotopic patterns, and comparison with databases and literature addressing *Nicotiana tabacum *metabolites; only signals consistently exhibiting an *s/n *of 10 or better are listed. ^1^Possible gas phase adducts.

### Alkaloids and phenolics

**Figure 3:**
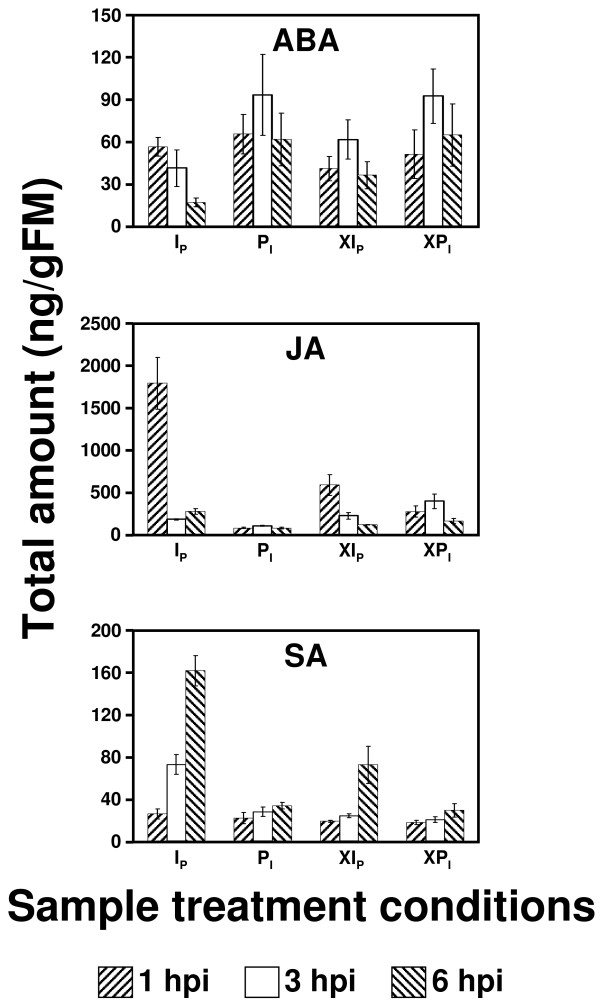
**Quantitative LC-ESI MS/MS analysis of phytohormones (time-profiles)**. Absolute amount of the major signaling phytohormones (ABA, JA, and SA) per weight of fresh leave material in two different cultivars of Tobacco (*SNN*, and *Xanthi*), determined by LC-ESI MS/MS in multiple reaction mode (MRM). Samples labeled with a preceding "X*" represent samples from *N. tabacum *(cv. *Xanthi*), * represents the sample treatment (for detailed explanation of the nomenclature employed, see text).

The alkaloid *tetrahydrobiopterin *(THB, Figure 2A) was identified as a metabolite for which the concentration increases substantially exclusively during local infection. THB functions as redox-cofactor for a number of metabolic processes (*e.g*. the oxidation of phenylalanine). In animals, it is also a cofactor of nitric oxide synthase (NOs) [47], but the exact plant metabolism of NO, an important messenger in stress response, is still controversial [48]. *Spermidine *(Figure 2B) a polyamine involved in various cellular processes [49], exhibits a moderate transient concentration increase (at ~ 3 hpi) in infected samples, but due to its multiple functions in plant metabolism it cannot be regarded a key indicator of infection. In contrast, *nicotine *levels (and possibly also those of its isomer anabasine) significantly decrease in infected leaf samples (Figure 2C). The alkaloid nicotine, a highly effective insecticide, is produced in the tobacco roots, transported to leaves, and stored in vacuoles. Increase of nicotine concentration in tobacco leaves has been related to the JA signaling cascade in response to attack from herbivores [4,29,50]. However, in contrast to the response to herbivore attack, infection with *Phytophthora nicotianae *results in a steep decline in nicotine concentration, when compared to placebo and blank samples. This metabolic change might be correlated with an increase in SA levels, an antagonist of JA (see hormone analysis below; Figure 3).

Phenolic metabolites whose biosynthesis is derived from the shikimic acid pathway [1,51], exhibit an increase in concentration during infection. Examples are two decarboxylation products of phenylalanine: *tyramine *(Figure 2D) and *phenylethylamine *(data not shown); *chorismic acid *(data not shown), a product of the shikimic pathway and major precursor of plant phenolics; and *chlorogenic acid *(data not shown), a derivative of coumaric acid. These observations concur with studies reporting a substantial up-regulation of the oxidative pentose-phosphate (OPP) pathway, which creates precursors for the shikimic pathway, and the L-phenylalanine-ammonia-lyase (PAL) activity, a major control step of the phenolic metabolism [17]. *Chorismic acid *or/and cinnamic acid (the product of the PAL reaction) are potential precursors of the hormone SA [1]. SA levels strongly increase after infection with *P. nicotianae *(Figure 3; see hormone analysis below).

### Free fatty acids and oxylipins

Concentrations of free fatty acids (FAs) in plant tissues, such as γ-*linolenic acid *(γ-LA, Figure 2E), *α-linolenic acid *(data not shown), and *palmitic acid *(data not shown), can be altered by wounding or infection [52-55]. For example, various oxylipins which function as stress messengers [52,54,55] are created by non-enzymatical or enzymatical (lipoxygenases) oxidation of FAs, in particular γ-LA [56-60]. Using IR-LDI-oTOF MS, three oxylipins were detected: *peroxidated *γ*-linolenic acid *(HOO-γ-LA, Figure 2F), *peroxidated methyl-*γ*-linolenate *(data not shown), and possibly *peroxidated chloride-methyl-α-linolenate *(data not shown); all are presumably derived from γ-LA [52,60]. High levels of LA's, in particular of γ-LA (Figure 2E), are only observed in pathogen-infected samples and may reflect activation of phospholipases. For example, phospholipase A2 (PLA_2_) is known to be activated in plants by infection [61] and constitutes the first step in the biosynthesis of the hormone JA. Increased levels of JA are indeed transiently found in infected samples at 1 hpi (see below; Figure 3). The decrease in HOO-γ-LA (Figure 2F) and JA abundances (while γ-LA increases) in locally and not-locally infected samples, which were not under mechanical-stress (such as B_I _samples), most likely reflects the infection - induced inhibition of pathways employed for lipid oxidation. This assumption is supported by the fact that the decrease in oxylipins and JA levels was not observed in samples that experience pure local mechanical-stress (which is known to activate the JA pathway). Moreover, the SA signaling cascade has been reported to block the first step in the lipoxygenase (LOX) cascade, which is responsible for the biosynthesis of JA from its precursor γ-LA [8,52].

### Carbohydrates

Recently, it was demonstrated for the tobacco - Phytophthora system that during development of the HR sugars (hexoses and dihexoses) accumulate in the cell wall [17,20]. Using IR-LDI oTOF MS, hexoses and dihexoses are detected in both positive and negative ionization modes. The time profiles of the potassiated sugar molecules are shown in Figure 2G & 2H, respectively. Although isomers cannot be differentiated in the MS profile, glucose and fructose are expected to comprise the major hexoses and sucrose the major dihexose in plants. In agreement with earlier biochemical analysis [17], the MS analysis indicates a significant increase in sugar levels (in particular for the dihexose) in infected samples.

### Plant hormones

ABA, a regulator of various environmental stress responses [62-64], positively affects the early (pre-invasion) defence [65]. It also influences the effect of the volatile hormone ET (which regulates the antagonistic signaling cascades of SA and JA) [66], and inhibits late defence reactions (such as the SA-mediated HR) [67]. The levels of *phaseic acid *(Figure 2I), the first stable decomposition product of ABA [68,69], increased exclusively in the infected samples, reflecting the possible degradation of ABA (see below, Figure 3)

Because IR-LDI MS ion signals of SA, JA, and ABA were either too low in intensity (SA) or not detectable (JA, ABA), we extracted these hormones from the plant lamina and used ESI-LC MS/MS to determine their absolute concentration in tissue [28-30]. The metabolic time profiles of the three hormones are displayed in Figure 3. The figure includes data obtained from two different cultivars of *Nicotiana tabacum*, the resistant cultivar *SNN *and the nonresistant cultivar *Xanthi*, which exhibits no or only a weak HR. In *SNN*, high JA levels appear transiently during the first hour post infection [70]. The rapid decline in JA after its initial burst (which is most likely due to the inhibition of the LOX cascade of the JA biosynthesis pathway) is possibly reflected in the IR-LDI-oTOF mass spectra by the "cross-over" between γ-LA (for which signals increase) and its peroxidation products (for which signals decrease). In contrast to JA, the levels of the phenolic hormone SA, known to inhibit JA biosynthesis [8,52], increase monotonously with time after injection of zoospores. The increase in SA concentration in the tobacco - Phytophthora system after 1 hpi is further supported by the appearance of PR1, a marker for the SA pathway [17]. In the LDI MS profile, infection-induced biosynthesis of SA may be reflected by the appearance of its phenolic precursors *chorismic acid *and *chlorogenic acid *(data not shown) [1,12]. In *Xanthi*, the initial JA burst was small and the increase in SA levels was retarded, compared to those seen in *SNN*, reflecting the difference in their response against the pathogen.

### Principal component analysis

**Figure 4:**
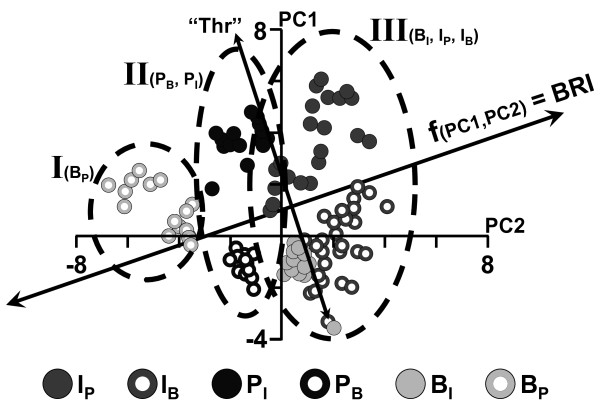
**Principal component analysis of the IR-LDI-oTOF MS spectra**. PC1 *vs*. PC2 score plot of an unsupervised PCA of the positive ion mode IR-LDI mass spectra acquired from differently treated samples of *Nicotiana tabacum *(cv. *SNN*). Based on their distribution with respect to the PC2-axis, the samples can, qualitatively, be grouped into three clusters: (I) untreated samples (blank near a placebo zone, B_P_); (II) "stressed" samples from local water infiltration (placebo near blank or infection zone; P_B_, P_I_); and (III) "stressed" samples from infection (I_P_, I_B_, B_I_). The line labeled as "Thr" separates samples that do not undergo HR (-HR) from those that undergo HR (+HR) (*i.e*. top right = high cell death, bottom left = low cell death). The line f(PC1,PC2) is displayed as the perpendicular line to "Thr". The "stress status" (BRI value, displayed in Figure 5) for each sample is obtained from the projection of any point to f(PC1,PC2).

**Figure 5:**
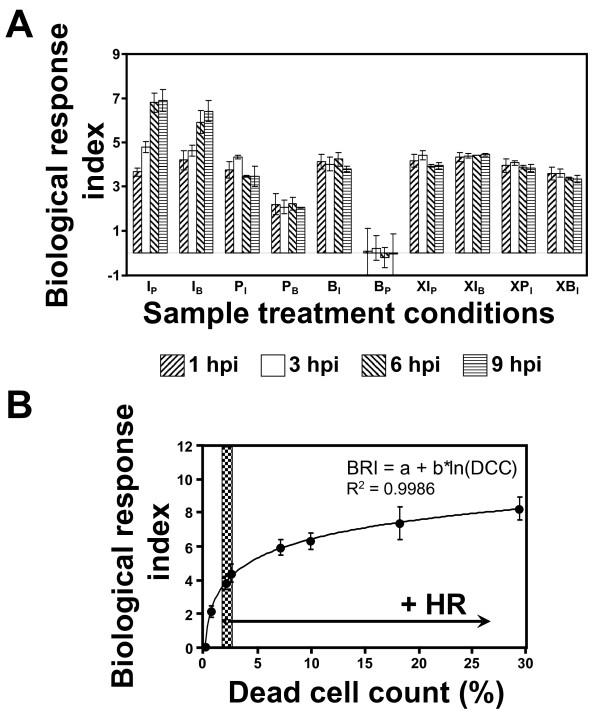
**Biological response index and comparison with dead cell count values**. (A) Biological response index (BRI, f(PC1,PC2)) obtained from the PCA shown in Figure 4 (see text for detailed explanation of the procedure). Samples labeled with a preceding "X*" represent samples from *N. tabacum *(cv. *Xanthi*), * represents the sample treatment (see text for details). (B) Comparison between BRI and the dead cell count (DCC, %) determined for differently treated *SNN *samples. Solid line: Best fit according to BRI = a + b*Ln(DCC), where a = 2.6044 and b = 1.6535 (R^2 ^= 0.9986). The shaded area indicates the threshold in terms of DCC that defines a positive HR.

A PCA transformation can help to visualize the most significant differences in the mass profiles between samples and allows similar samples to be grouped together. The first principal component (PC1) is most strongly influenced by the combination of ion signals that exhibit the largest change between the recorded spectra. In a similar way, subsequent principal components (PC2, PC3, etc) represent less influential changes that can, however, still help to identify particular features of the investigated system. Figure 4 shows the PC1 *vs.* PC2 score plot of the unsupervised PCA performed with the positive mode ion spectra (see Material and Methods for details). This graphic representation reveals a high correlation between the MS profiles of the six different groups of investigated samples. Based on PC2 values, they are essentially grouped into one of three "qualitative" clusters. Qualitatively, these clusters may be seen as representing: (I) untreated samples (blank near a placebo zone, B_P_); (II) "stressed" samples from local water infiltration (placebo near blank or infection zone; P_B_, P_I_); and (III) "stressed" samples from infection (I_P_, I_B_, B_I_). This qualitative analysis suggests that PC2 may capture both systemic and local stress signals, though the local stress response is presumably reflected stronger. In the score plot, samples are not differentiated with respect to the time post treatment. As can be seen in Figure 5, mass spectra of the infected samples tend to show an increase of the PC1/PC2 values derived from their metabolic profile with time post infection, while the picture for the other sample treatments is less clear.

### Identification of key metabolites and their pathways

The corresponding loading plots (displayed in Additional file 2) identify which metabolites, and therefore their associated pathways, contribute the most to the variance of the two principal components. Some of these metabolites can directly be related to known metabolic processes. Examples for PC1: *phaseic acid *(*m/z *281.140, *cf*. to Table 1 for the type of ion formation) relates to ABA turnover (see hormone analysis above); *tyramine *(*m/*z 138.091, *cf*. to Table 1) relates to the phenolic metabolism and SA turnover (see hormone analysis above). Examples for PC2: HOO-γ-LA (*m/z *311.220, *cf*. to Table 1) and its methyl ester (*m/z *325.236, *cf*. to Table 1) relate to LOX activity and JA turnover (see hormone analysis above). The loading plots also revealed MS signals that exhibit a high correlation in the PCA, for which the identity of the corresponding ions, however, is currently unknown. For example, the ion signal with an *m/z *value of 318.153 (Table 1) shows a strong correlation with PC1. Based on its mass, this metabolite could possibly be either a glycosylated ester of the alkaloid *retronecin *[71], or a gas phase adduct of protonated *tyramine + *hexose (or both). A second signal with an *m/z *value of 361.211 (Table 1) shows a strong correlation with PC2. This species might be peroxidated-chlorinated-*α*-methyl-linolenate or a gas phase adduct of protonated *peroxidated-methyl-γ-linolenate *with hydrochloric acid. Further studies are needed to elucidate the chemical identity of these species. These examples demonstrate that the defence response of tobacco (*SNN*) toward *Phytophthora Nicotianae *can only be more fully captured by the combination of the two main principal components (PC1 & PC2).

### Biological response index

Because only locally infected tissue of the *SNN *cultivar undergoes HR, while neighboring leaf areas do not exhibit HR, two qualitatively lines can be drawn in the PC1 *vs*. PC2 score plot of Figure 4. The first one, labeled as "Thr" (threshold), is a line that separates all measured samples from tobacco (*SNN*) into two regions: (a) +HR (undergoing HR) and (b) -HR (not undergoing HR), respectively. The second one is perpendicular to "Thr", and is labeled as f(PC1,PC2). The projection of the PC1 *vs*. PC2 data points onto this line - f(PC1,PC2) - can be regarded as reflecting the "stress status" of the samples. One may thus call this one-dimensional projection an integrated "Biological Response Index" (BRI).

Figure 5A shows the so-derived BRI values for the differently treated *SNN *and *Xanthi *samples for time points between 1 to 9 hpi. For convenience, the mean of the BRI values derived for "untreated" samples (B_P_), where a minimum of metabolic effects can be expected to occur, was furthermore arbitrarily set to zero. The highest BRI values of ~7 were derived for infected *SNN *samples at 6 and 9 hpi, while infected *Xanthi *samples exhibit intermediate values of ~4, which are essentially constant at all time points, and also similar to the BRI values obtained for P_I _and B_I _*SNN *samples.

To further evaluate the possible relevance of the BRI, we examined how well the BRI values correlate with the number of cells that had undergone HR by performing classical dead cell count analysis of identically treated samples. The correlation between the two values is plotted in Figure 5B. Cell death is a consequence of (SA - mediated) HR and proportional to the strength of the plant defence response. The BRI *vs*. DCC plot (Figure 5B) reveals a non-linear function of the form "a + b*Ln(DCC)" between the qualitative BRI and the quantitative DCC values. The non-linearity of the relationship may be interpreted as describing the complex metabolic (and hormonal) transition, starting from initial local or "micro-systemic" stress or infection responses to a specific defence reaction. BRI values around 5 reflect the transition from more or less reversible stress response to irreversible processes (such as HR) which inevitably result in cell death (represented by the line labeled "Thr" in Figure 4), but this transition is not abrupt.

The BRI parameter appears to only reflect the strength of the local defence response toward the infection. Treatments applied on the opposite side of the midvein did not notably influence the BRI value obtained for the infected samples. This demonstrates that at least within the time frame of 1 - 9 hpi, the analysis of leaf sections which develop a HR will not be dramatically affected by major systemic effects which traversed the major veins (Additional file 3). However, the occurrence of more minor systemic effects, that are influencing the overall metabolic profile, is well reflected by the original PCA score plot. For example, in the PC1 *vs*. PC2 score plot of Figure 4, the metabolic profile of blank control samples is placed adjacent to an infection site, suggesting that PC2 may capture "micro-systemic" metabolic responses.

**Figure 6:**
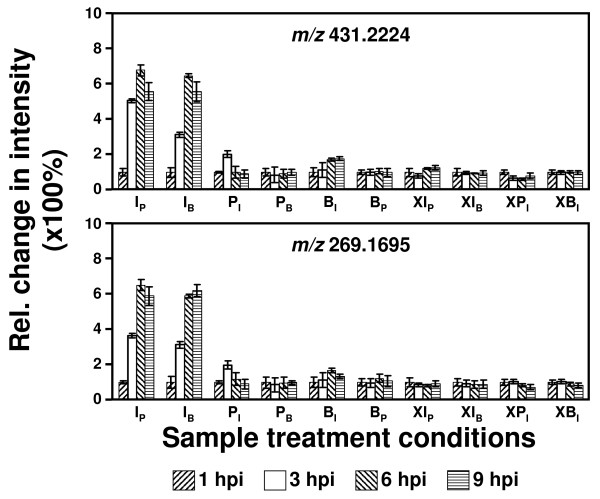
**Putative biomarkers for the HR of tobacco (*SNN*)**. Metabolic time-profiles (determined by direct IR-LDI-oTOF MS) of two, as yet unidentified metabolites exhibiting *m/z *values of 431.222 and 269.170 that exhibit a strong correlation with the biological response index (BRI, f(PC1,PC2)) and could be employed for monitoring the defence response of *N. tabacum *(cv. *SNN*) toward *P. nicotianae *infection.

Employing loading plots (Additional file 2) to find those metabolites that are associated with a high BRI value, two ion species with *m/z *values of 269.170 and 431.222, respectively, were identified to exhibit a particular high correlation with the BRI. The metabolic time profiles of these, as yet unidentified compounds, are displayed in Figure 6. Both species essentially show the same expression profile with time post treatment. The mass difference of 162.052 u suggests that these two metabolites likely represent the deglycosylated/glycosylated derivative of each other, where the sugar moiety would be a hexose. The strong correlation with the BRI value and thus to the infection-related stress response renders them as putative biomarkers for monitoring the HR defence response. Future studies will aim to structurally characterize the two compounds.

## Conclusions

IR-LDI-oTOF mass spectrometry allowed the rapid and simultaneous detection of a wide range of naturally occurring (unlabeled) metabolites directly from leaf lamina samples of tobacco plants. Changes in time for the signals of secondary metabolites (such as phenolics, alkaloids, and oxylipins) and primary metabolites (such as hexoses and dihexoses) were recorded for differently treated samples. The recorded changes in the metabolome indicated that the JA/SA-defence cascades as well as the ABA turnover cascade were involved in stress/infection response. The involvement of these three cascades can explain the majority of the physiological changes that were previously observed during this plant - pathogen infection [17,20]. The hypothesis that the JA/SA-defence cascades as well as ABA turnover are important for the development of HR was successfully verified by LC-ESI-MS/MS analysis of phytohormones.

From the PCA of the positive ion mode spectra a qualitative stress response factor (denominated: biological response index, BRI) was derived. By employing the PCA loading plots, several ion signals were identified to be strongly correlated with the local defence response against the pathogen. While many of these metabolites have been described previously to be involved in stress/infection response, two ion signals that are showing a particular strong correlation with the BRI could so far not be assigned. Potentially, the two compounds can serve as biomarkers for monitoring the HR. Thus, IR-LDI-oTOF MS analysis in combination with PCA may help to not only to rapidly record complex metabolic transitions during specific stress responses, but also to identify such potential biomarkers.

Furthermore, two additional advantages of the LDI-oTOF MS method are that it first allows to record metabolic profiles directly from fresh tissue and second provides a high lateral resolution. This simplifies and accelerates the analysis, on the one hand, and facilitates the mapping of the spatial spread of the metabolic response across the leaf on the other. Recent developments even indicate the possibility to analyze individual plant cells [39]. Thus, in nearby future; this method could be beneficial for studying developmental processes, which exhibit distinctive spatial orientation as well as several types of stress responses. In the case of the defence of the *N. tabacum *cultivar *SNN *toward *P. nicotianae*, the HR is strictly a localized process, however the defence response may not need to be (there might be signals going out which prepare neighboring cells to execute HR faster). Hence, future high resolution metabolome mapping could be an extremely powerful tool, e.g. for detecting the spread of an infection and for analyzing the interaction between infected and non-infected cells.

## Materials and methods

### Chemicals

Deuterated abscisic acid (D_2_-ABA) and jasmonic acid (D_2_-dihydro-JA) were purchased from OlChemIm Ltd (Olomouc, Czech Republic); deuterated salicylic acid (D_4_-SA), and all other chemicals/solvents were purchased from Sigma-Aldrich (Steinheim, Germany) and were used without further purification.

### Plant and pathogen material

Plants were grown in a climate chamber with 24°C and 22°C (day and night temperature, respectively), with a 14 hour photoperiod (light levels of 300 μmol quanta·m^-2^·s^-1^). Source leaves of six to eight weeks old plants from two cultivars of *Nicotiana tabacum *were used. Because only about two source leaves of this age are found in an individual tobacco plant, several identically treated plants were employed for this study. The cultivar *SNN *is resistant against the employed pathogen and capable of developing a strong HR; the other cultivar, *Xanthi*, is susceptible and does not produce sizable HR (thus, it was employed as a biological reference) [17]. Cultivation of *Phytophthora nicotianae *van Breda de Haan isolate 1828 (DSMZ, Braunschweig, Germany), the production of zoospores, and the infection procedure were performed as described previously [17,20].

### Preparation of samples for IR-LDI-oTOF MS

Three types of samples were collected from plant leaves: Blank samples (B) were from untreated leave areas, placebo samples (P) from areas which got sterile tap water injected to represent the pure injection stress, and infected samples (I) which where injected a suspension of 750 ± 250 zoospores per μl. In case of the P and I samples, several injections of either tap water or zoospore suspension were placed close to each other within an area of ~20 cm^2^; the total injected volume per sample was 1 ml. The liquid was slowly infiltrated with a fine syringe to fill the air space in the *spongy mesophyll *of the leaves. The benefit of employing *Phytophthora nicotianae *(which initiates local HR responses in leaves of resistant tobacco) as a pathogen and using an artificial infection procedure (local infiltration of leaves) is that the infection area is well defined and the mesophyll cells respond nearly synchronously. To determine whether, besides the local reactions of interest, there are also systemic effects, combinations of the three treatments were applied to three sets of leaves. One specific treatment was applied on one side of the midvein and the second was applied to the other. Subscripts identify this treatment; for example, I_p _would be a sample taken from a zoospore-injected site with a placebo (*i.e*. water injection) on the opposite side of the midvein.

For each treatment, injections were placed on a given side of the leaf in areas, where the different time points were separated by side veins (*i.e*. one distal zone for each time point) in order to minimize crosstalk between different samples [72]. Typically, three discs of 7.5 - 15 mm in diameter were cut out of each treatment area and analyzed by IR LDI-oTOF MS. The punch sites were all placed as close as possible to the point of injection. A sketch illustrating the example of a leave with spore injection on the right side of the leaf and a blank of the left is shown in Figure 1.

Samples were shock-frozen in liquid nitrogen immediately after dissection, and subsequently stored at -80°C until the MS measurements. Immediately prior to the IR-LDI MS measurement, and while the leaf was still frozen, the lower cuticle of the leaves was removed using adhesive tape. Leaf samples were placed on a standard microscope glass slide "upside down" (*i.e*. the lower epidermis side of the leaf was "face-up") using adhesive (G304, Plano W. Plannet GmbH, Wetzlar, Germany) (Figure 1). The glass slide was mounted into a custom-made MALDI sample plate using double-sided tape.

To visualize the changes in the metabolic profile that are specifically induced by infection from those initiated by mechanical or osmotic stress (due to leaf infiltration or wounding by sample collection) IR-LDI mass spectra acquired from pathogen-infiltrated (infected) leaf sections were compared with those recorded from water-infiltrated (placebo), and untreated (blank) leaf sections. Furthermore, to minimize the local variations in the amount of zoospores present in the tissue, mass profiles were taken from the middle of the injection zones. A sketch illustrating this procedure is provided in Additional file 4.

Samples were collected at time points of 1, 3, 6, and 9 hpi. Typically, the injected water exuded/evaporated within 45 min. Because a low mass spectral quality was obtained from plant tissue containing excessive water in the air space of the spongy mesophyll,1 hpi was chosen as the earliest measurement time point. By comparing changes of the relative intensity of an ion signal determined at the different time points, normalized to its intensity at the reference point of 1hpi, "key metabolites" that exhibit significant changes with time post treatment can be identified. Care was taken that samples did not reside inside the fine vacuum of the MS ion source longer than 10 min to avoid excessive water loss by dehydration.

### IR-LDI-oTOF MS analysis of metabolites

The mass spectrometer has been described in detail before [34,40]. This instrument is equipped with an UV-laser (N_2 _laser, *λ *= 337 nm) and an Er:YAG laser (Speser, Spektrum, Berlin, Germany). The latter is an IR-laser that emits pulses of ~150 ns duration at a wavelength of 2.94 μm and at a repetition rate of 2 Hz. The IR-laser beam irradiated the leaf surfaces at an angle of incidence of 45°. The focal spot size was slightly elliptical and had an area of ~150 × 200 μm^2 ^(1/e^2 ^intensity definition), the beam profile was near-Gaussian. N_2 _was used as buffer gas for collisional cooling and focusing [44]. The pressure in the sample region of the ion source was adjusted to ~0.7 mbar [46]. For ion generation, a laser fluence (energy per pulse and area) of ~25 kJ·m^-2 ^was typically applied. At this fluence, tissue layers of about 50 μm in depth are removed per laser pulse in the central part of the focused beam, allowing the analysis of the content of lysed cells. 120 laser shots where accumulated for each single spectrum. These successive exposures were applied over an area of typically 2 mm^2 ^by manually moving the sample target under the fixed laser beam in a zigzag pattern. This way, any given sample location was exposed to no more than four laser pulses, thereby avoiding a full penetration of the sample. Two mass spectra were recorded from each individual sample disk.

Measurements were performed in positive and negative ion mode using time-of-flight voltages of ± 10 kV. The mass resolution (FWHM) was about 6,000 in both ion modes. Using the monoisotopic ion signals of Angiotensin I ([M+H]^+ ^*m/z *1296.6853) and 2,5-dihydroxybenzoic acid (DHB) UV-MALDI matrix molecules ([M+H]^+ ^*m/z *155.0344) for external calibration, a mass accuracy of about ~20 ppm was achieved for the *m/z *range between 100 - 500. All ions were detected as singly charged species. With internal calibration, using signals of metabolites of known identity (*e.g*. nicotine, hexose, and dihexose), the mass accuracy could be improved to ~6 ppm. Mass spectra were partially processed using MoverZ software (freeware edition, Genomics Solution, Ann Arbor, MI, USA).

Although HR signals are locally triggered in the pathogen infection area [17,21], plants possess the ability to generate disease resistance against further infections in healthy (uncompromised) parts by employing secondary metabolites as infection messengers. As shown in Additional file 4A, the location from where the sample disk was taken within the same distal zone may to some extent influence the metabolic pattern in the mass spectra, due to changes in the local concentrations of zoospores in the tissue. To account for this source of variability, all sample disks were taken from the center of the placebo or infected areas located inside one distal zone and as equidistance as possible to the multiple points of injection. In the case of the blank samples, the sample disks were taken from the center of their respective distal zones (Additional file 4B).

If the samples were taken from the center of their respective distal zones, the main parameters that influenced the relative signal intensities and ion patterns were the amount of water contained in the leaves and the applied laser fluence (see above, preparation of samples for IR-LDI-oTOF MS). Attempts to improve a quantitative data evaluation by finding an endogenous "internal standard" were not successful. Therefore, care was taken to follow a consistent preparation and measurement protocol. Under these conditions, the variation in the intensities of specific metabolite signals obtained from identically prepared and treated samples (from the same or different plants) was between 5 - 30%. This is illustrated by the error bars in the graphs.

### Identification of metabolites

Table 1 lists the presumable chemical composition and type of ion formation for all ~60 consistently detected ion species, in positive (Table 1A) and negative (Table 1B) ion mode, along with the experimental and calculated *m/z *values. Positively charged ions are mostly detected as protonated [M+H]^+ ^and partially as sodiated/potassiated [M+Na]^+^/[M+K]^+ ^species, negatively charged ions as deprotonated [M-H]^- ^and partially as chlorinated molecules [M+Cl]^- ^(identified by the distinct ^35^Cl/^37^Cl isotopic distribution of chlorine).

In addition, a few gas phase adducts such as [hexose + hexose + K]^+ ^and [tyramine + hexose + H]^+ ^are presumably also detected due to the soft desorption/ionization conditions [40]. Since the oTOF instrument does not provide a tandem MS option for structural characterization of ions, isobaric species can not be differentiated. Hence, putative identification of metabolites is based on the comparison of experimental and theoretical mass values (which had to coincide within 20 ppm), the comparison of experimental and theoretical isotopic patterns, and the use of various databases addressing general plant metabolites, as well as literature addressing *Nicotiana tabacum *metabolism. The following databases were used in this study: **ChemBioFinder **http://chembiofinder.cambridgesoft.com/; **Chemspider **http://www.chemspider.com/; **Kegg **http://www.genome.jp/kegg/; **LipidBank **http://lipidbank.jp/; **LipidMaps **http://www.lipidmaps.org/; **Metlin **http://metlin.scripps.edu/**Plant metabolomic pathway **http://plantcyc.org/; and **Welmetser **http://maths.sci.shu.ac.uk/students/bioinformatics/Bindal_Nidhi/WELMETSER.pl.

### Principal component analysis

The oTOF mass spectra were converted to ASCII files using the custom-made data acquisition software of the mass spectrometer (TOFMA v99.6; developed by W. Ens and colleagues at the University of Manitoba, Winnipeg, CA). The ASCII files were converted into Microsoft excel sheets using a home-made program to become compatible with the PCA program RapidMiner 4.600 (Rapid-I, Dortmund, Germany). Only ion signals that were consistently detected with a signal-to-noise (*s/n*) ratio of at least 10 at any of the monitored time points for *SNN *plants were used for the unsupervised PCA. Because the focus of this study was on the resistant *SNN *cultivar, those ions which were unique to *Xanthi *and not seen in *SNN *with the necessary *s/n *ratio were excluded from the PCA.

### Preparation of samples for LC-ESI MS

LC-ESI MS quantitation of selected metabolites (phytohormones) was performed based on the work published by the Baldwin group (Max Planck Institute for Chemical Ecology in Jena, Germany) [28-30]. The infection procedure was identical to the one employed for the IR-LDI-oTOF MS measurements. Eight disks of 0.9 mm in diameter were cut out with a puncher. Samples were shock-frozen in liquid nitrogen immediately after dissection and subsequently stored at -80°C in 2 ml vials. Free phytohormones were extracted by homogenizing ~100 mg of leaf material in a SamplePrep 2000 Geno/Grinder (SPEX CertiPrep, Metuchen, NJ, USA), employing FastPrep tubes containing 900 mg of lysing matrix (BIO 101; Vista, California, USA) and 1 ml of ethyl acetate spiked with 100 ng of deuterated internal standards D_4_-SA and D_2_-ABA; and 40 ng of D_2_-dihydro-JA. Phytohormones were extracted by reciprocal shaking at 6.5 m s^-1 ^for 2 min; consecutively, they were centrifuged at 13,000 rpm for 20 min at 4°C. The supernatants were evaporated to dryness in a vacuum concentrator at 30°C. The dry residue was re-dissolved in 600 μl of methanol (70%) and centrifuged at 13,000 rpm for 10 min at 4°C. An aliquot (500 μl) was transferred to HPLC vials for measurement.

### LC-ESI MS/MS analysis of phytohormones

The transferred aliquots were quantified using an LC-ESI tandem mass spectrometry system (Varian 1200, Palo Alto, USA). 10 μl of each extracted sample were injected onto a ProntoSIL column (C18; 5 μm, 50 × 2 mm; Bischoff, Leonberg, Germany) attached to a precolumn (C18, 4 × 2 mm; Phenomenex, Aschaffenburg, Germany). The mobile phase comprised solvent A (0.05% formic acid) and solvent B (methanol) used in a gradient mode (time/concentration [min/%] for B: 0.0/15; 1.5'/15; 4.5'/98; 12.5'/98; 13.0'/15; 15.0'/15) with a flow of (time/flow [ml/min]: 0.0'/0.4; 1.0'/0.4; 1.5'/0.2; 10.0'/0.2; 10.5'/0.4; 15.0'/0.4). Compounds were detected as negative ions in multiple reaction monitoring (MRM) mode. Molecular ions [M-H]^- ^at *m/z *137, 209, 263 (SA, JA, and ABA, respectively) and 141, 211; 265 (deuterated derivatives of the compounds, respectively) were fragmented using 15 eV collision energy. The ratios of ion intensities of non-deuterated and deuterated product ions were used to quantify endogenous SA, JA, and ABA.

### Dead cell count analysis

The infection procedure was identical to the one employed for the IR-LDI-oTOF MS measurements. Dead cell count was performed with 0.5 mg ml^-1 ^propidium iodide aqueous solution based on the work published by the Weis group (Institute of Botany, Westfälische Wilhelms-Universität Münster, Germany) [21]. Cells with disrupted membranes (dead cells) allow the fluorescence dye propidium iodide to enter into the cytoplasmatic region, thus indicating cell death. The fluorescence was detected at 590 - 650 nm after excitation at 488 nm using a confocal laser scanning microscope (TCS SPE2 with inverse DM IRB-microscope, Leica, Wetzlar, Germany) [21].

## List of abbreviations

2,5-DHB: (2,5-Dihydroxybenzoic acid); ABA: (Abscisic acid); ASCII: (American standard code for information interchange); B_I_: (Sample taken from an untreated site with an infected, *i.e*. spore-injected, on the opposite side of the midvein); B_P_: (Sample taken from an untreated site with a placebo, *i.e*. water injection, on the opposite side of the midvein); BRI: (Biological response index); D_2_-ABA: (Deuterated abscisic acid); D_2_-dihydro-JA: (Deuterated dihydro-jasmonic acid); D_4_-SA: (Deuterated salicylic acid); DCC: (Dead cell count); DSMZ: (German collection of microorganisms and cell culture - Deutsche Sammlung von Mikroorganismen und Zellkulturen GmbH); Er:YAG: (Erbium-doped yttrium aluminum garnet); ESI: (Electrospray ionization); ET: (Ethylene); FA: (Free fatty acid); FM: (Fresh mass - weight of fresh leaf); GC: (Gas chromatography); γ-LA: (γ-linolenic acid); HOO-γ-LA: (Peroxidated γ-linolenic acid); hpi: (Hours post infiltration - hours post infection); HR: (Hypersensitive response); I_B_: (Sample taken from a spore-injected site with a blank, *i.e*. untreated, on the opposite side of the midvein); I_P_: (Sample taken from a spore-injected site with a placebo, *i.e*. water injection, on the opposite side of the midvein); IR: (Infrared); JA: (Jasmonic acid); LA: (linolenic acid); LC: (Liquid chromatography); LDI: (Laser desorption/ionization); LOX: (Lipoxygenase); MALDI: (Matrix assisted laser desorption/ionization); MRM: (Multiple reaction monitoring); MS: (Mass spectrometry); MS/MS: (Tandem mass spectrometry); N_2_: (Molecular nitrogen); NO: (Nitric oxide); OPO: (Optical parametric oscillator); OPP: (Oxidative pentose phosphate pathway); oTOF: (Orthogonal time-of-flight); PAL: (L-phenylalanine-ammonia-lyase); P_B_: (Sample taken from a water injection site with a blank, *i.e*. untreated, on the opposite side of the midvein); PC: (Principal component); PCA: (Principal component analysis); P_I_: (Sample taken from a water injection site with an infected, *i.e*. spore-injected, on the opposite side of the midvein); PLA_2_: (Phospholipase A2); PR1: (Pathogenesis-related gene 1); ROS: (Reactive oxygen species); *s/n*: (Signal-to-noise ratio); SA: (Salicylic acid); SNN: (Samsun cultivar of *Nicotiana tabacum*); THB: Tetrahydrobiopterin); TOF: (Time-of-flight).

## Competing interests

The authors declare that they have no competing interests.

## Authors' contributions

AJI conceived the study, carried out the IR-LDI MS and statistical analysis, and drafted the manuscript. JS, PB, AP, SM, ITB, FH, EW, and KD participated in the design and coordination of the study, and contributed to the manuscript. JS and PB grew the plants and bacteria, and carried out the plant infections. JS performed the death cell count analysis. AP wrote the data mining program. SM and AJI carried out the LC-ESI MS/MS analysis of phytohormones. All authors read and approved the final manuscript.

## Supplementary Material

Additional file 1 **IR-LDI-oTOF MS spectra**. Representative IR-LDI-oTOF mass spectra showing metabolic profiles obtained in positive and negative ion modes from an I_P _and a P_I _*SNN *tobacco leaf sample at 6 hpi. Presumable chemical compositions are identified for selected major ions species. A full list of detected ion signals and their tentative identities is provided in Table 1. Click here for file

Additional file 2 **Unsupervised loading plots generated from the PCA**. Loading plots of the PCA result of Figure 4, showing all positive ions listed in Table 1; for improved clarity, nominal mass values are displayed. Ions of particular interest are highlighted in boldface, including phaseic acid (*m/z *281.140) as predominantly associated with the ABA turnover, three oxylipins at *m/z *311.220, 325.236, and 361.211, and the phenolic metabolite tyramine (*m/z *138.091) from the shikimic pathway. Metabolites that display the highest correlation to the biological response index (BRI), and consequently to the strength of the infection are shown in *italic* Click here for file

Additional file 3 **Validation of the BRI index**. The BRI reflects the local defence response. Comparing the BRI plot and the dead cell count (DCC, %) in *SNN *obtained from infected samples with different sample treatment environments on the opposite side of the midvein (I_B_: blank; I_P_: placebo; I_I_: infected). For the calculation of the BRI, 3 plant per infection times 2 leaves per plant times 3 samples per plant were used; for the DCC, 3 plants per infection times 3 leaves per plant times 14 Infection sites per plant and 17 cLSM pictures per infection site were employed. Click here for file

Additional file 4 **IR-LDI-oTOF MS analysis of metabolites (spatial-profile)**. Metabolic profiles detected by direct IR-LDI-oTOF of Nicotiana tabacum (cv. *SNN*) leaf samples from one single distal zone measured at 6 hpi. (A) Mass spectra were acquired from 7.5 mm diameter sample disks located within the infection zone. (B) Mass spectra acquired by successively moving the sample target, hence the area irradiated by the IR laser, across the imaginary border between healthy and infection zones present in one distal zone of the leaf. Click here for file
