# Supplementary material for: Rapid metabolic profiling of Nicotiana tabacum defence responses against Phytophthora nicotianae using direct infrared laser desorption ionization mass spectrometry and principal component analysis
Source: Plant Methods. 2010 Jun 9;6:14. doi: 10.1186/1746-4811-6-14 (PMC2904756; doi:10.1186/1746-4811-6-14)
Supplement: Additional file 1 — IR-LDI-oTOF MS spectra. Representative IR-LDI-oTOF mass spectra showing metabolic profiles obtained in positive and negative ion modes from an IP and a PI SNN tobacco leaf sample at 6 hpi. Presumable chemical compositions are identified for selected major ions species. A full list of detected ion signals and their tentative identities is provided in Table 1. [file 1746-4811-6-14-S1.PDF]

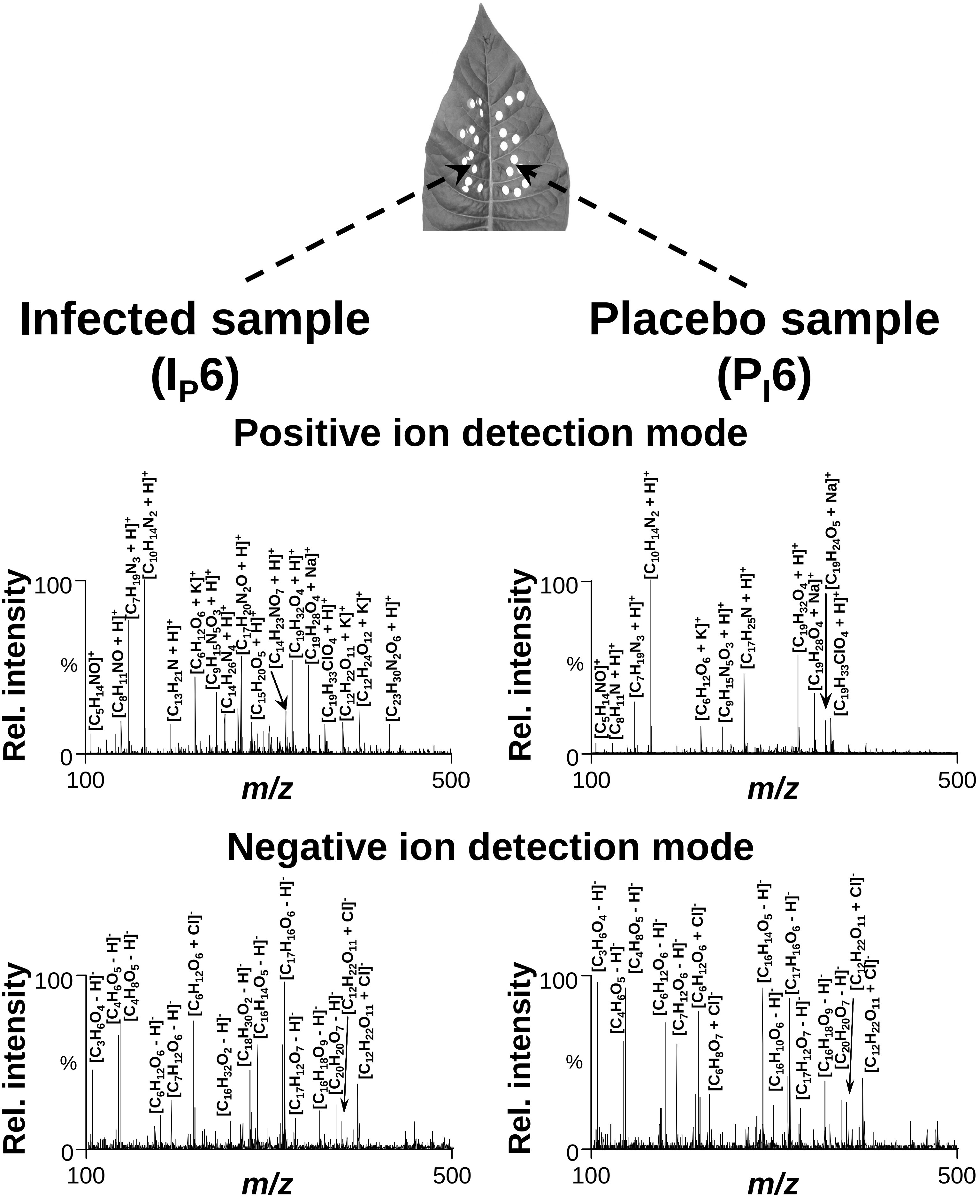

Infected sample
(IP6)
Placebo sample
(PI6)
Positive ion detection mode
Negative ion detection mode
Rel. intensity
%
100
0
100
500
m/z
[C5H14NO]+
[C8H11NO + H]+
[C7H19N3 + H]+
[C10H14N2 + H]+
[C13H21N + H]+
[C6H12O6 + K]+
[C9H15N5O3 + H]+
[C14H26N4 + H]+
[C17H20N2O + H]+
[C15H20O5 + H]+
[C14H23NO7 + H]+
[C19H32O4 + H]+
[C19H28O4 + Na]+
[C19H33ClO4 + H]+
[C12H22O11 + K]+
[C12H24O12 + K]+
[C23H30N2O6 + H]+
[C8H11N + H]+
[C17H25N + H]+
[C19H24O5 + Na]+
[C3H6O4 - H]-
[C4H6O5 - H]-
[C4H8O5 - H]-
[C6H12O6 - H]-
[C7H12O6 - H]-
[C6H12O6 + Cl]-
[C16H32O2 - H]-
[C18H30O2 - H]-
[C16H14O5 - H]-
[C17H16O6 - H]-
[C17H12O7 - H]-
[C16H18O9 - H]-
[C20H20O7 - H]-
[C12H22O11 + Cl]-
[C6H8O7 + Cl]-
[C16H10O6 - H]-
