# Supplementary figures and images for: Rapid metabolic profiling of Nicotiana tabacum defence responses against Phytophthora nicotianae using direct infrared laser desorption ionization mass spectrometry and principal component analysis
Source: Plant Methods. 2010 Jun 9;6:14. doi: 10.1186/1746-4811-6-14 (PMC2904756; doi:10.1186/1746-4811-6-14)

# PC2 Loading

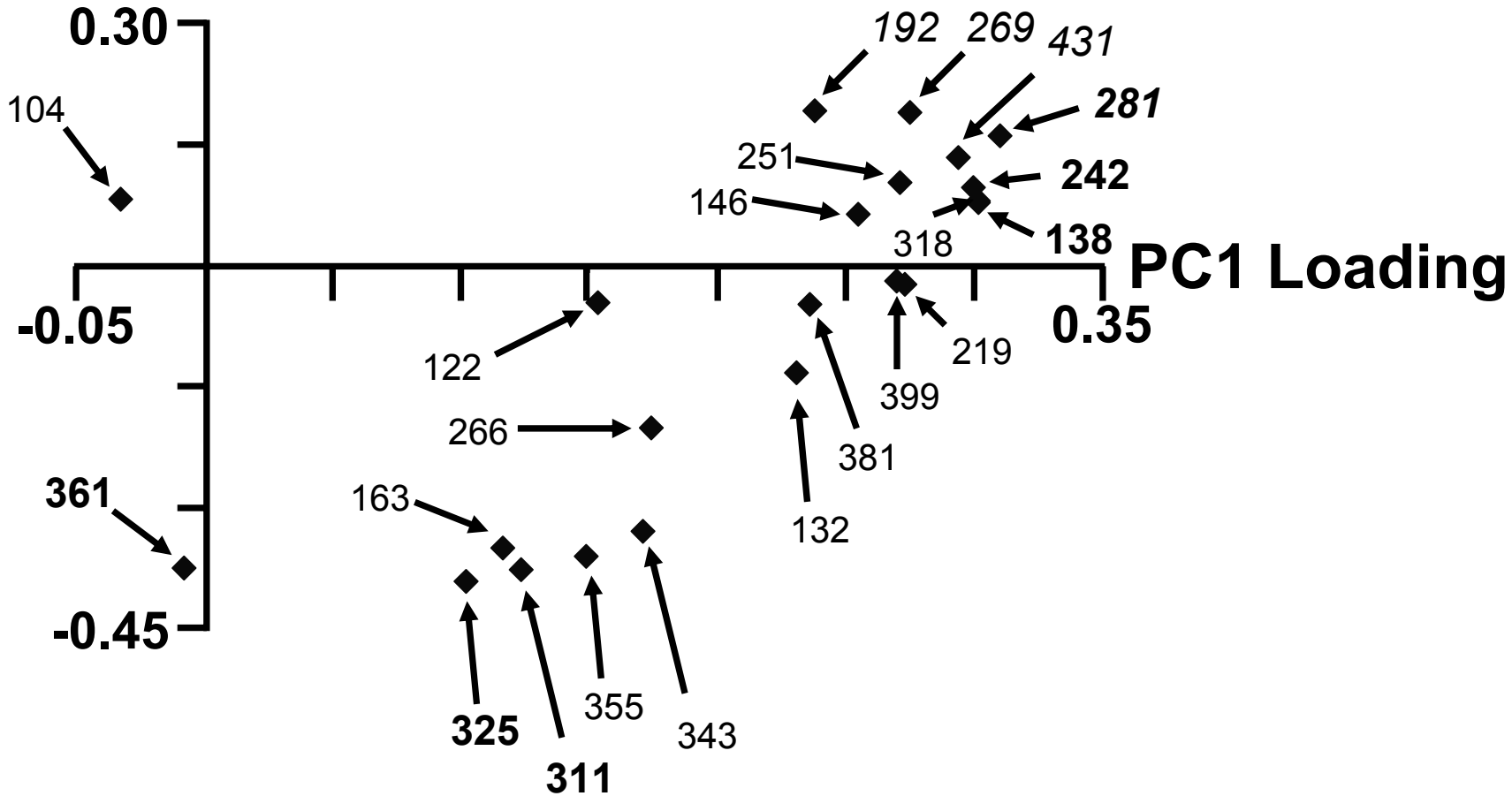

Supplement: Additional file 2 — Unsupervised loading plots generated from the PCA. Loading plots of the PCA result of Figure 4, showing all positive ions listed in Table 1; for improved clarity, nominal mass values are displayed. Ions of particular interest are highlighted in boldface, including phaseic acid (m/z 281.140) as predominantly associated with the ABA turnover, three oxylipins at m/z 311.220, 325.236, and 361.211, and the phenolic metabolite tyramine (m/z 138.091) from the shikimic pathway. Metabolites that display the highest correlation to the biological response index (BRI), and consequently to the strength of the infection are shown in italic [file 1746-4811-6-14-S2.PDF]

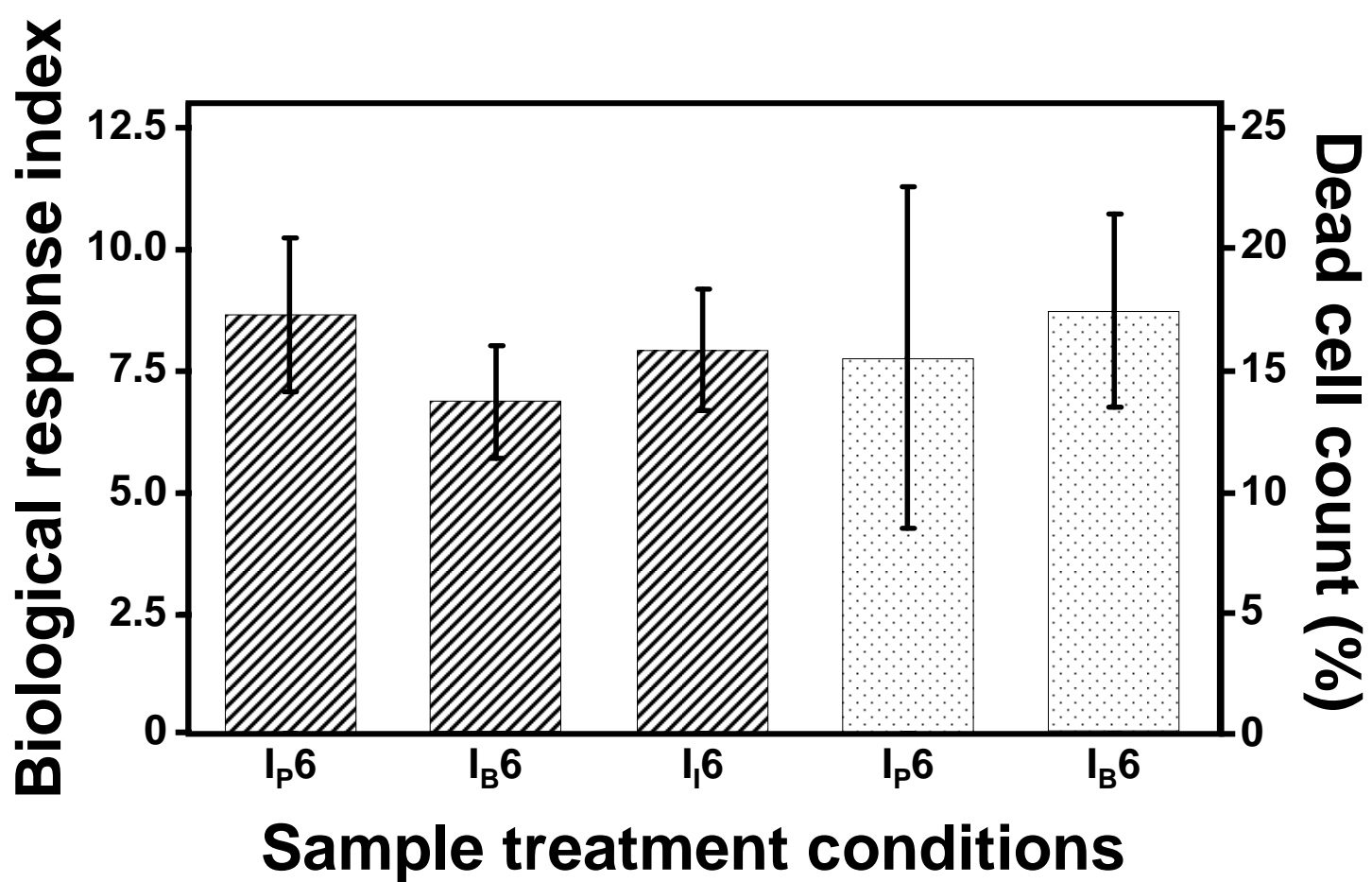

Supplement: Additional file 3 — Validation of the BRI index. The BRI reflects the local defence response. Comparing the BRI plot and the dead cell count (DCC, %) in SNN obtained from infected samples with different sample treatment environments on the opposite side of the midvein (IB: blank; IP: placebo; II: infected). For the calculation of the BRI, 3 plant per infection times 2 leaves per plant times 3 samples per plant were used; for the DCC, 3 plants per infection times 3 leaves per plant times 14 Infection sites per plant and 17 cLSM pictures per infection site were employed. [file 1746-4811-6-14-S3.PDF]

**A**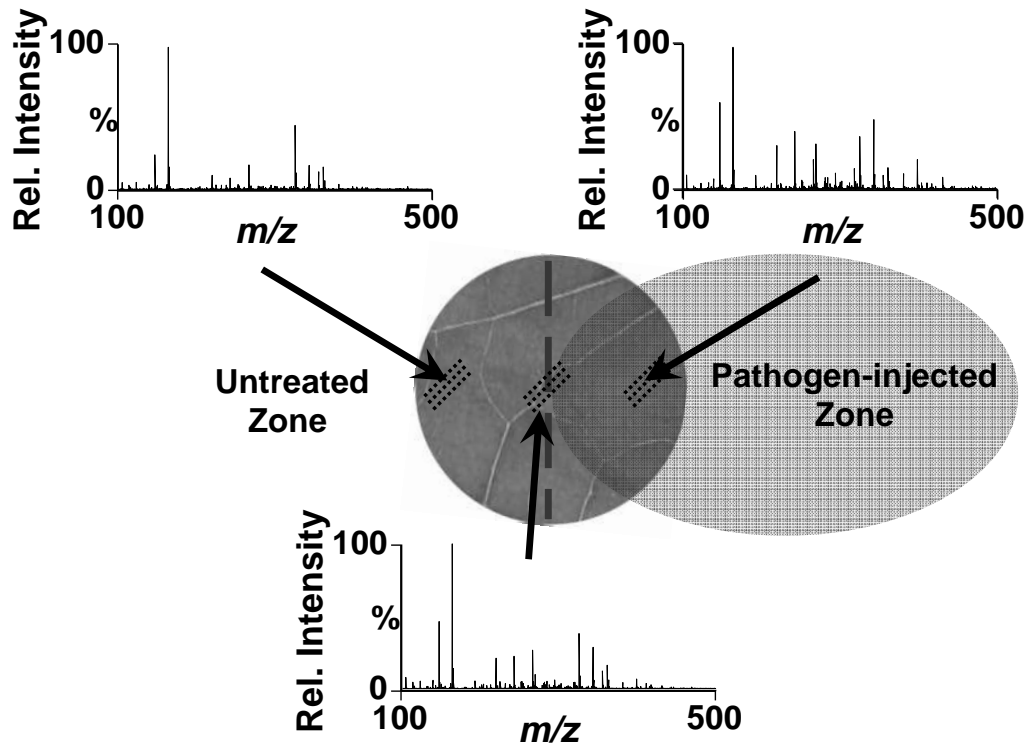**B**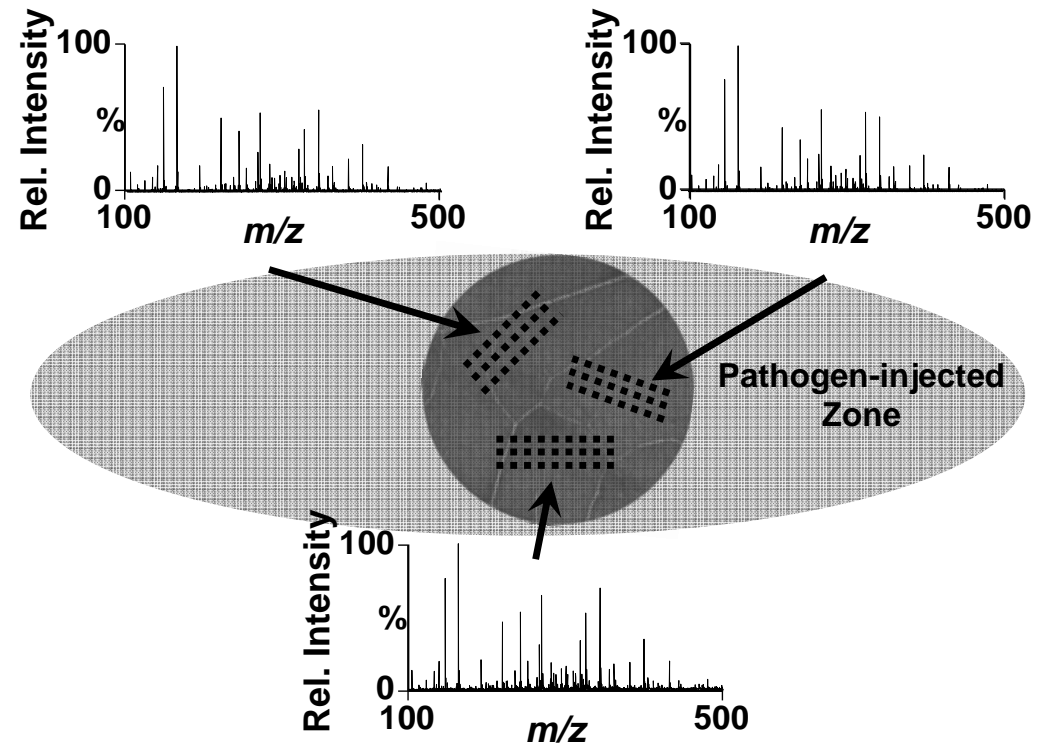

Supplement: Additional file 4 — IR-LDI-oTOF MS analysis of metabolites (spatial-profile). Metabolic profiles detected by direct IR-LDI-oTOF of Nicotiana tabacum (cv. SNN) leaf samples from one single distal zone measured at 6 hpi. (A) Mass spectra were acquired from 7.5 mm diameter sample disks located within the infection zone. (B) Mass spectra acquired by successively moving the sample target, hence the area irradiated by the IR laser, across the imaginary border between healthy and infection zones present in one distal zone of the leaf. [file 1746-4811-6-14-S4.PDF]
